# The relationship between self-rated health and local government spending on parks and recreation in the United States from 1997 to 2012

**DOI:** 10.1016/j.pmedr.2018.11.018

**Published:** 2018-12-07

**Authors:** J. Tom Mueller, So Young Park, Andrew J. Mowen

**Affiliations:** a Department of Agricultural Economics, Sociology, and Education, College of Agricultural Sciences, The Pennsylvania State University, United States of America; b Department of Recreation, Park, and Tourism Management, College of Health and Human Development, The Pennsylvania State University, United States of America

**Keywords:** Health promotion, Environment, Leisure activities, Policy making, Recreation

## Abstract

The purpose of this study was to analyze the relationship between local government spending on parks and recreation and self-rated health in the United States. Using four publicly available datasets from the U.S. Census Bureau, the Current Population Survey, the Decennial Census, the American Community Survey, and the State and Local Government Finance Survey for the years 1997–2012 (n = 303,203), we estimated a multinomial and a binary logit model predicting self-rated health with county area percentage of expenditures contributed to parks and recreation operations as the independent variable of interest. A one-percent increase in the portion of county area expenditures contributed to parks and recreation operations was associated with decreased relative risk of very good (RRR = 0.95; 95% CI = 0.93, 0.96), good (RRR = 0.95; 95% CI = 0.93, 0.97), or fair (RRR = 0.89; 95% CI = 0.87, 0.92) health relative to excellent health. The effect held in the binary logit model for adult men and women, but not youth. Higher levels of parks and recreation spending were associated with higher levels of self-rated health for adults across the United States from 1997 to 2012. Investing greater portions of local government budgets in parks and recreation operations may have the potential to improve self-rated health among residents.

## Highlights

• Higher parks and recreation spending was associated with better self-rated health.
• The relationship held for adult men and women, but not youth.
• Increasing contributions to parks and recreation may improve public health.

## 1 Introduction

Demonstrating the relationship between government spending and tangible outcomes is essential for evidence-based policy making. With regard to the impact of local government spending on parks and recreation on individual health outcomes, the evidence remains scarce and inconclusive (Rosenberger et al., 2005; Humphreys and Ruseski, 2007). While literature concerning the relationship between spending and health outcomes is limited, evidence linking the use and availability of parks and recreation services to positive health outcomes has been consistent (Rosenberger et al., 2009; Cohen et al., 2015; Cohen et al., 2007; Mullenbach et al., 2018; Pitas et al., 2017a; Hughey et al., 2016; Bedimo-Rung et al., 2005; Zarr et al., 2017). Parks and recreation services have been linked to lower levels of obesity across the United States and within West Viriginia (Rosenberger et al., 2005; Rosenberger et al., 2009), higher levels of physical activity in California and across the United States (Cohen et al., 2015; Cohen et al., 2007; Mullenbach et al., 2018), and higher levels of self-rated health in Pennsylvania and South Carolina (Pitas et al., 2017a; Hughey et al., 2016). Additionally, there is evidence that many individuals view parks as an essential component of the healthcare system (Mowen et al., 2017). Given the demonstrated association between parks and recreation services and health outcomes, we extended previous research by examining the direct relationship between local government parks and recreation spending and individual level self-rated health. If local government spending on parks and recreation has a direct effect on public health, it carries significant implications for policy priorities.

Following its peak prior to the Great Recession in 2007, local government spending on parks and recreation was on the decline until 2014 (Crompton and Kaczynski, 2003; Pitas et al., 2017b). Although local government spending on parks and recreation has rebounded, it is still below pre-recession levels, and is outpaced by the recovery of other government services (Pitas et al., 2017b). This decline is contrasted by the consistent increase of healthcare spending in the United States, with current projections expecting the United States to spend 19.9% of its gross domestic product (GDP) on healthcare by 2025 (Centers for Medicare and Medicaid Services, 2017). This increase, which continued throughout the Great Recession, even when overall GDP was declining, has been the subject of criticism and concern, including reports of diminishing returns from increased direct healthcare spending (Murphy and Topel, 2003).

As local government parks and recreation funding ebbs and flows with the national economy, it is important to understand how increases or decreases in funding may impact public health. To do this, we test the hypothesis that increases in the portion of the budget a county area contributed to parks and recreation over the period of 1997 to 2012 would be associated with a decrease in the relative risk of an individual reporting more negative levels of self-rated health over that time period. Self-rated health has been established as a simple, but reliable, marker of holistic health throughout the literature due to its strong and consistent relationship with mortality (Phillips et al., 2005; Jylhä, 2009; Blakely et al., 2001).

We contribute to the literature by evaluating the impact of government spending on self-rated health across the United States over a 15-year period. To do this, we capitalized on an existing linkage between self-rated health and county level identification available in the U.S. Current Population Survey (CPS) by combining it with the U.S. State and Local Government Finance Survey (SLGFS), the decennial U.S. Census, and the American Community Survey (ACS) five-year estimates. Data linking individual-level health markers to the geographic scale of county is relatively rare and, to our knowledge, there is currently no other research available examining the impact of parks and recreation spending on individual level health at a similar temporal or spatial scale.

## 2 Methods

### 2.1 Data sources and variables of interest

To conduct our analysis, we used four datasets: the SLGFS, the U. S. CPS, the decennial U.S. Census, and the ACS five-year estimates (Historic County Area Finance Data, State, and Local Finance Census, 2011; County Area Finance Data, 2007; County Area Finance Data, 2012; Flood et al., 2017; Manson et al., 2017). The SLGFS is conducted by the U.S. Census Bureau and collects information on all government revenue and expenditures from the local to the federal level. The SLGFS conducts a full census of all U.S. governments in years ending in 2 and 7 and expenditure data is categorized into a wide variety of spending types (U.S. Census Bureau, 2006). For our analysis, we extracted expenditure data at the county area level, which is different from spending at simply the county level. County area spending represents all government expenditures on a given category coming from within a county. This includes expenditures from the county government plus all local governments within that county. This aggregation is preferable due to its ability to more appropriately and accurately capture local government spending on parks and recreation occurring with a county as well as allowing us to include non-standard counties in our analysis such as the five boroughs of New York City, which are treated as a larger ‘New York City’ county area, and the parishes of New Orleans which are treated as independent county areas.

The CPS data was generated from the Integrated Public Use Microdata Series – CPS dataset (IPUMS-CPS) (Flood et al., 2017). This dataset is a publicly available microdata series with >50 years of data. The CPS is the U.S. monthly labor force survey, and included in this survey is information at the individual level concerning race, income, employment, gender, and many other demographic variables. Beginning in 1996, the March Annual Social and Economic Supplement of the CPS also contained self-rated health. CPS data, while not representative at the county level, does contain county level identifiers. Due to concerns of confidentiality, counties with low representation are not identified within IPUMS-CPS. This results in approximately 45% of cases being identified at the county level, meaning this sample is largely made up of residents living in counties with above average population. As the data were collected from different samples for each March survey supplement, and not all counties were present in every year, this dataset is pooled cross-sectional.

Given that the SLGFS only collects data in years ending in 2 and 7, and that self-rated health was not available in the CPS until 1996, we extracted four years of observations for our analysis from the SLGFS and the CPS: 1997, 2002, 2007, and 2012 (Historic County Area Finance Data, State, and Local Finance Census, 2011; County Area Finance Data, 2007; County Area Finance Data, 2012). County level control variables from the decennial U.S. Census and the ACS five-year estimates were extracted from the National Historical Geographic Information Systems database hosted by IPUMS (IPUMS-NHGIS) (Manson et al., 2017). Due to the removal of the long-form census from the 2010 census, ACS five-year estimates for 2006–2010 were used for our 2010 county level demographic control variables of education and income. Given that the county level demographic data needed for our control variables were historically only collected in decennial years, we extracted two years of county level demographic data, 2000 and 2010, and assigned the data for those years to the year ending in 7 preceding the decennial year and the year ending in 2 following the decennial year.

The datasets were then merged, with each individual case being assigned the expenditure data and county level control variables from their corresponding county. Where necessary, time-consistent geographic county units were created by collapsing counties into larger geographic areas where county boundaries may have changed during the study period (Dwyer-Lindgren et al., 2016). This data was ruled as not human subjects research by our institutional review board.

### 2.2 Variables

#### 2.2.1 Dependent variable

The dependent variable in our analysis was self-rated health. Respondents to the CPS were asked, “Would you say your health in general is excellent, very good, good, fair, or poor?” This measure is coded as 5 = Poor, 4 = Fair, 3 = Good, 2 = Very Good, 1 = Excellent. We analyzed the variable as both a five-point scale, as well as a binary variable of ‘poor’ or ‘good’ health. In this case the self-rated health variable was recoded as either ‘poor’ or ‘good’, where ‘poor’ was made up of respondents selecting either Poor or Fair and ‘good’ was made up of respondents selecting Good, Very Good, or Excellent.

#### 2.2.2 Independent variable of interest

In order to evaluate the association between local government parks and recreation spending on self-rated health we used the portion of overall county area expenditures contributed to parks and recreation operations from the SLGFS. County area parks and recreation operational spending was then divided by total county area spending and multiplied by 100 to create a variable representing the percentage of their budget a county area contributed to parks and recreation operations. The use of a proportional measure, as opposed to strict dollars, restricts the possible influence that localized governmental wealth may have on self-rated health. According to the U.S. Census, parks and recreation is the “provision and support of recreational and cultural-scientific facilities maintained for the benefit of residents and visitors.” (U.S. Census Bureau, 2006). SLGFS provides an aggregate level of data for the category of parks and recreation that includes the following items: “…golf courses, playgrounds, tennis courts, public beaches, swimming pools, playing fields, parks, camping areas, recreational piers and marinas, etc.; galleries, museums, zoos, and botanical gardens; auditoriums, stadiums, recreational centers, convention centers, and exhibition halls; community music, drama, and celebrations including public support of cultural activities.” (U.S. Census Bureau, 2006).

We used operational expenditures, as opposed to overall or capital expenditures, for two reasons: First, operational expenditures are relatively stable year to year and include maintenance, upkeep, staffing, and many other costs (U.S. Census Bureau, 2006) making them a more appropriate measure of a local government area's investment in parks and recreation than capital spending, which is likely to be more inconsistent and occur as sporadic large-scale investments. Second, operational expenditures capture government investment in parks and recreation programs, a dimension of parks and recreation previously shown to impact self-rated health (Pitas et al., 2017a).

#### 2.2.3 Control variables

We included individual-level control variables known to influence self-rated health including ethnicity/race, education level, household income, age, and sex (Phillips et al., 2005). Ethnicity/race was collapsed into four categories and dummy coded into three variables, Hispanic, non-Hispanic Black, and non-Hispanic other, with non-Hispanic White as the reference group. Education was collapsed into five categories and four dummy variables: less than a high school diploma, high school diploma, some college, college graduate, and graduate degree holder. The reference group for the education dummy variables was less than a high school diploma. Age was in years and was included as a linear and quadratic effect due to an expected non-linear relationship across the range of ages. Household income was total household income in dollars and was logarithmically transformed due to a non-normal distribution. The quadratic of the logged term was also included due to the likelihood of a non-linear relationship. Sex was dummy coded as 1 = female and 0 = male. We included a variable to control for the possibility that healthy people may have moved into a county recently coded as 1 = lived within the county during the past year or 0 = moved into the county in the past year.

Six county level demographic control variables expected to influence both health and county level spending were also included in order to avoid bias due to confounding variables: percentage of population with a bachelor's degree or higher, logarithmically transformed median income, median age, percent non-Hispanic White, percent non-Hispanic Black, percent Hispanic. Additionally, three forms of county-level spending expected to have an association with health and parks and recreation spending were included to avoid bias: the proportion of total county area expenditures contributed to health, welfare, and hospitals. Finally, we included fixed effects in the form of dummy variables for state and year to add geographic and temporal controls to our model.

### 2.3 Data analysis

Two models were estimated using percent of county area operational spending on parks and recreation as the independent variable of interest. First, a multinomial logistic regression was performed with five-level self-rated health as the dependent variable, using Excellent self-rated health as the baseline. Second, a binary logistic regression predicting ‘poor’ health was estimated. Both models used Stata/MP version 14.0. Initially, we employed the ordered logit model but our data violated the Brant test of the proportional odds assumption, which is one of the key assumptions to utilize the ordered logit model. This violation is common (Long et al., 2014), and we elected to use a multinomial logistic regression since we intended to compare each category in a binary manner (Williams, 2016). The Stata command mlogit with robust estimator of variance, vce(r), was used for the multinomial logistic regression. For the coefficients, the relative risk ratio with 95% confidence intervals are reported.

We conducted two sensitivity tests, the inclusion of a youth (17 and under) variable and the inclusion of county level fixed effects, wherein you treat each county as its own control and only look at within-county variation. The inclusion of the youth variable did not impact the model, meaning no significant (*p* < .05) associations in the independent variable of interest became non-significant. When we included county level fixed effects the impact of parks and recreation spending on self-rated health became non-significant (*p* ≥ .05). However, we believe this was due to the limited variation in spending at the county level. As the maximum variation within counties was only four observations, and there was zero within-year individual variation in the independent variable due to the hierarchical nature of the data, we believe the inclusion of county level fixed effects significantly limited the power of the analysis to detect an effect. Therefore, we feel it is important to note, but we do not feel it diminishes or casts doubt on the validity of our findings. Finally, we also compared the results from the multinomial logit model to the same model ran as a multinomial probit regression and found minimal differences.

In addition to our multinomial logit model, we performed a binary logistic regression predicting ‘poor’ health (Fair or Poor self-rated health), a method similar to previous research using self-rated health (Blakely et al., 2001; Blakely et al., 2002). Given the relatively few people in the sample responding as Poor on the five-point scale (n = 8892), the use of the binary model allowed us to assess the association with a larger number of people in the ‘poor’ group. The same independent variables were included in this analysis as in the multinomial logistic regression and robust standard errors were used. We present this model for overall, adult women, adult men, and youth, in order to show the consistency of the relationship across different segments of the population.

## 3 Results

Summary statistics for independent and dependent variables are provided in Table 1. Our sample included a total of 303,203 people and represented 316 counties in 40 states. Our hypothesis, that we would observe an inverse relationship between higher levels of parks and recreation operational spending and poorer levels of self-rated health, was supported in all categories of the multinomial logit model except for the model from excellent to poor (Table 2). The percentage of county area operational spending on parks and recreation had a significant (*p* < .05) association with individuals rating their health as Excellent as opposed to Very Good, Good, or Fair. Compared to the baseline of Excellent self-rated health, a 1% increase in the portion of overall expenditures contributed to parks and recreation operations was associated with an average decrease in the relative risk of respondents falling into the category of Fair, Good, or Very Good health by 0.89, 0.95, and 0.95 respectively (Table 2). That is, those who lived in a county that contributed a larger portion of their budget to parks and recreation operations were more likely to report themselves in Excellent self-rated health. Additionally, spending on parks and recreation was the only spending variable included in the model with a significant (*p* < .05) negative impact on the likelihood of reporting poorer levels of self-rated health throughout all categories.

**Table 1:** Descriptive statistics for variables included in models.

Variable	Mean	Std. dev.	Min	Max
Individual-level characteristics				
Self-rated health^a^	2.12	1.06	1.00	5.00
Age (years)	34.19	21.79	0.00	90.00
Age^2^ (years)	1643.56	1712.77	0.00	8100.00
Sex (proportion male)	0.52	0.50	0.00	1.00
Race (proportion of total)				
Non-Hispanic White	0.56	0.50	0.00	1.00
Non-Hispanic Other	0.07	0.26	0.00	1.00
Non-Hispanic Black	0.14	0.34	0.00	1.00
Hispanic	0.24	0.42	0.00	1.00
Education (proportion of total)				
Less than high school	0.41	0.49	0.00	1.00
High school	0.21	0.40	0.00	1.00
Some college	0.14	0.34	0.00	1.00
College	0.18	0.39	0.00	1.00
Graduate school	0.07	0.25	0.00	1.00
Migration status^b^	0.94	0.23	0.00	1.00
Family income (log dollars)	10.52	1.80	0.00	14.26
Family income^2^ (log dollars)	113.91	26.45	0.00	203.38
N	303,203			
County-level characteristics				
Median income (log dollars)	11.04	0.25	10.30	11.78
Median age (years)	35.68	3.40	23.30	54.80
Education level (%)^c^	18.91	6.13	6.12	49.46
Population (per 10,000)	158.15	244.60	0.00	997.49
Non-Hispanic White (%)	59.48	21.38	3.40	97.75
Non-Hispanic Black (%)	14.03	12.57	0.17	66.50
Hispanic (%)	19.88	18.02	0.38	95.74
Current operational spending (% of total expenditures)				
Parks and recreation	1.68	0.90	0.18	6.94
Health	2.66	2.17	0.00	16.46
Welfare	3.43	4.25	0.00	24.49
Hospital	4.03	6.93	0.00	47.28
N (counties)	316			

a Self-rated health is coded as 5 = Poor, 4 = Fair, 3 = Good, 2 = Very Good, 1 = Excellent.

b Migration status is given the value 1 if the respondent resided in the same house or only moved within county in the past year, and the value 0 if the respondent either moved within state to a different county, between states, or abroad.

c Percentage of population that holds a bachelor's degree is used as a proxy for average education level of a county.

**Table 2:** United States county level current operational spending on parks and recreations and self-rated health 1997–2012.

	4 (very good)^a^, ^b^, ^c^			3 (good)			2 (fair)			1 (poor)		
	N = 95,947			N = 70,446			N = 21,931			N = 8892		
	RRR (SE)	CI	*p*	RRR (SE)	CI	*p*	RRR (SE)	CI	*p*	RRR (SE)	CI	*p*
Current operational spending (county level, % of total expenditures)												
Parks and recreation	0.95 (0.01)	0.93–0.96	<0.001	0.95 (0.01)	0.93–0.97	<0.001	0.89 (0.02)	0.87–0.92	<0.001	0.96 (0.02)	0.92–1.00	0.05
Health	1.00 (0.00)	1.00–1.01	0.51	1.00 (0.00)	1.00–1.01	0.55	1.00 (0.01)	0.99–1.01	0.98	1.00 (0.01)	0.98–1.02	0.98
Welfare	1.00 (0.00)	1.00–1.01	0.17	1.01 (0.00)	1.00–1.01	0.03	1.01 (0.00)	1.01–1.02	0.01	1.00 (0.01)	0.99–1.02	0.73
Hospital	1.00 (0.00)	1.00–1.00	0.02	1.00 (0.00)	1.00–1.00	0.04	1.00 (0.00)	0.99–1.00	0.08	1.00 (0.00)	0.99–1.00	0.10

Note: Multinomial logistic regression; CI = 95% confidence interval; RRR = relative risk ratio. Robust standard errors in parentheses. The baseline for all models is Excellent self-rated health (n = 105,987). Overall N = 303,203. For the sake of clarity and space we only present an abbreviated table here, a more comprehensive table is available in the Appendix.

a Individual level controls for age, age^2^, sex, race, household income, household income^2^, and migration status were included in the model but are not presented in this table due to space.

b County level median level income, median age, population, percent Hispanic, percent non-Hispanic Black, percent non-Hispanic White, and percent with a bachelor's degree were included as controls but are not presented here.

c State and year dummies were included in the regression but were also excluded from the table due to space.

**Fig. 1:**
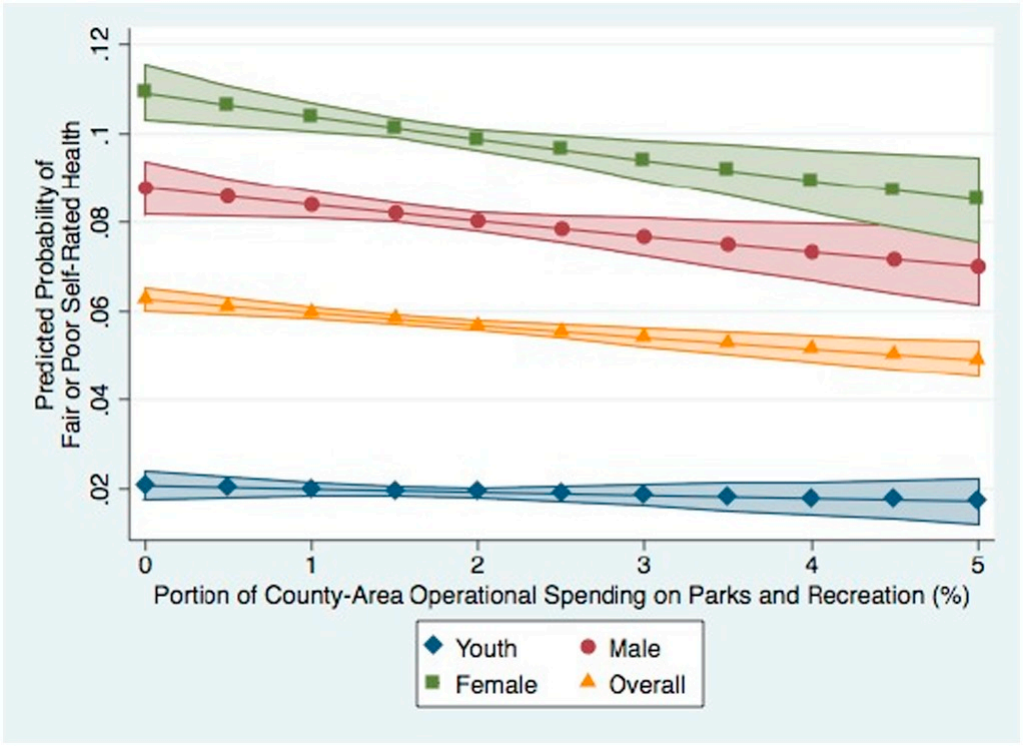
Predicted probability of Fair or Poor self-rated health given the portion of the United States county area spending contributed to parks and recreation (%) from 1997 to 2012, with all other variables held at their mean.

The results of the binary logistic regression predicting ‘poor’ health, where a value of 0 was given to the categories of Good, Very Good, and Excellent and 1 to those of Poor and Fair, were consistent with the results of the multinomial logistic regression. For all segments of the population we considered except for youth – adult men, adult women and overall – higher portions of county area expenditures contributed to parks and recreation operations were associated with a significant (*p* < .05) decrease in the relative risk of an individual reporting either Poor or Fair health, as opposed to Good, Very Good, or Excellent health (Table 3). The association between parks and recreation spending and self-rated health was stronger than for any other form of spending. Fig. 1 presents the predicted probabilities of a respondent reporting Poor or Fair health for each segment of the population while holding all other variables in the model at their mean. The negative trend-line for three of the four groups visually demonstrates the negative association that increased county area spending on parks and recreation had with the probability of individuals reporting Fair or Poor health throughout the study period.

**Table 3:** County level operational spending on parks and recreations and binary self-rated health, by age and sex 1997–2012.

	Overall^a^, ^b^, ^c^			Female adult			Male adult			Youth^d^		
	N = 303,203			N = 112,020			N = 99,251			N = 91,801		
	OR (SE)	CI	*p*	OR (SE)	CI	*p*	OR (SE)	CI	*p*	OR (SE)	CI	*p*
Current operational spending (county level, % of total expenditures)												
Parks and recreation	0.95 (0.01)	0.93–0.97	<0.001	0.95 (0.02)	0.91–0.98	0.00	0.95 (0.02)	0.92–0.99	0.02	0.96 (0.04)	0.88–1.05	0.41
Health	1.00 (0.00)	0.99–1.01	0.65	1.00 (0.01)	0.99–1.01	0.92	1.00 (0.01)	0.98–1.01	0.60	0.99 (0.02)	0.96–1.02	0.60
Welfare	1.01 (0.00)	1.00–1.01	0.11	1.01 (0.01)	1.00–1.02	0.08	1.00 (0.01)	0.99–1.01	0.81	1.01 (0.01)	0.98–1.03	0.50
Hospital	1.00 (0.00)	1.00–1.00	0.35	1.00 (0.00)	0.99–1.00	0.25	1.00 (0.00)	1.00–1.00	0.82	1.00 (0.00)	0.99–1.01	0.71

Note. CI = 95% confidence interval; OR = odds ratio. Robust standard errors in the parentheses. For the sake of clarity and space we only present an abbreviated table here, a more comprehensive table is available in the Appendix.

a Individual level controls for age, age^2^, sex, race, household income, household income^2^, and migration status were included in the model but are not presented in this table due to space.

b County level median level income, median age, population, percent Hispanic, percent non-Hispanic Black, percent non-Hispanic White, and percent with a bachelor's degree were included as controls but are not presented here.

c State and year dummies were included in the regression but were also excluded from the table due to space.

d The number of observations for youth decreased from 91,932 to 91,801 due to the omission of the observations from Arkansas, which had no observation for the ‘poor’ category.

## 4 Discussion

While prior research has indicated a positive relationship between park use and physical activity (Cohen et al., 2015; Cohen et al., 2007; Mullenbach et al., 2018), as well as recreation program participation and self-rated health (Pitas et al., 2017a; Hughey et al., 2016), little is known concerning the association between parks and recreation spending and public health outcomes. We used four publicly available census datasets to determine the relationship between county level parks and recreation spending and self-rated health from 1997 to 2012. Given the inconsistent level of funding for local parks and recreation relative to other government services (Pitas et al., 2017b), it is important to understand the potential impact of these spending decisions on public health.

We found, when controlling for relevant demographic characteristics, higher levels of county area parks and recreation spending were significantly associated with lower relative risk of reporting poorer levels of self-rated health, with the exception of the comparison from Excellent to Poor—suggesting that parks and recreation is associated with all but the most extreme changes in self-rated health. When we segmented the population by age and gender to explore the binary model of ‘good’ or ‘poor’ health, we found that higher levels of parks and recreation spending were associated with decreased relative risk of ‘poor’ self-rated health for adult women and adult men, but not for youth. Further, out of all included spending variables expected to associate with health, parks and recreation was the only variable associated with better self-rated health.

Our findings add to the growing body of literature highlighting the important role that parks and public spaces play in public health outcomes (Rosenberger et al., 2009; Cohen et al., 2015; Cohen et al., 2007; Mullenbach et al., 2018; Pitas et al., 2017a; Hughey et al., 2016; Bedimo-Rung et al., 2005; Zarr et al., 2017). Given the possible diminishing returns of increased levels of traditional forms of healthcare spending, evaluating the impacts and efficacy of alternatives is essential for evidence driven public health policy. The consistent relationship between spending and self-rated health in our models suggests prior research characterizing government funded parks and recreation services as a part of the health care system may be appropriate (Mowen et al., 2017). These findings add further credibility to the efforts of groups pushing for local park use as a form of preventive medicine (e.g. Park Rx) (Zarr et al., 2017). In light of these findings it appears that funding for local parks and recreation services could be viewed as a form of non-traditional healthcare spending. When viewing local parks and recreation services in this way, the inconsistent nature of their funding over the past fifty years is troubling (Crompton and Kaczynski, 2003; Pitas et al., 2017b), and may suggest the need for a reconsideration of priorities, both at the local government level and within public health policy.

### 4.1 Future research and limitations

As with any study, there are limitations to our analysis that should be addressed through further research. We highlight three here. First, while we have shown that increased levels of local government funding for parks and recreation were associated with decreased relative risk of lower levels of resident self-rated health, it is not the increase in funding that directly impacts health. Rather it is what that funding would support, provide, and encourage. Future research should attempt to link local government parks and recreation funding to park use and physical activity, and ultimately public health outcomes (e.g. preventing chronic disease).

Second, operational funding for parks and recreation contains many forms of spending. Thus, while it would be valuable to look at more specific forms of spending, the SLGFS does not report any further of level of disaggregation. In-depth qualitative case-studies of specific localities would help researchers gain a better understanding of what forms of parks and recreation funding impact localized health outcomes. Further, a quantitative understanding of how parks and recreation agencies spend money, and how that impacts public health is necessary.

While this sample is large and represents much of the United States, not every county is identified in the CPS due to risks of confidentiality. Of particular note are places with below average population (e.g. rural areas). Researchers should attempt to explore these relationships with greater geographic representation. Finally, the nature of our data prohibited the consideration of inequality. The benefits of parks and recreation spending are not likely to be felt by all. Future research should attempt to explore how these health benefits are distributed throughout the population.

### 4.2 Conclusion

In this paper we tested the hypothesis that higher portions of their budget that county areas contributed to parks and recreation over the period of 1997 to 2012 would be associated with lower relative risk of an individual reporting lower levels of self-rated health. Our findings generally support this hypothesis. When controlling for relevant individual, county, and state level characteristics, county areas that contributed a greater portion of their overall expenditures to parks and recreation operations from 1997 to 2012 had adult residents who were significantly less likely to report their self-rated health as either Fair or Poor. These findings suggest that local governments are likely to experience significant public health benefits by increasing their budgetary contribution to parks and recreation.

## Conflicts of interest

None.

## Figures and Tables

**Table 2:** United States county-level current operational spending on parks and recreations and self-rated health 1997–2012.

	4 (very good)			3 (good)			2 (fair)			1 (poor)		
	N = 95,947			N = 70,446			N = 21,931			N = 8892		
	RRR (SE)	95% CI	*p*	RRR (SE)	95% CI	*p*	RRR (SE)	95% CI	*p*	RRR (SE)	95% CI	*p*
Current operational spending (county level, %)												
Parks and recreation	0.95 (0.01)	0.93–0.96	<0.001	0.95 (0.01)	0.93–0.97	<0.001	0.89 (0.02)	0.87–0.92	<0.001	0.96 (0.02)	0.92–1.00	0.05
Health	1.00 (0.00)	1.00–1.01	0.51	1.00 (0.00)	1.00–1.01	0.55	1.00 (0.01)	0.99–1.01	0.98	1.00 (0.01)	0.98–1.02	0.98
Welfare	1.00 (0.00)	1.00–1.01	0.17	1.01 (0.00)	1.00–1.01	0.03	1.01 (0.00)	1.01–1.02	0.01	1.00 (0.01)	0.99–1.02	0.73
Hospital	1.00 (0.00)	1.00–1.00	0.02	1.00 (0.00)	1.00–1.00	0.04	1.00 (0.00)	0.99–1.00	0.08	1.00 (0.00)	0.99–1.00	0.10
Demographics												
Age, years	1.02 (0.00)	1.02–1.03	<0.001	1.04 (0.00)	1.04–1.04	<0.001	1.09 (0.00)	1.09–1.10	<0.001	1.15 (0.00)	1.14–1.16	<0.001
Age^2^	1.00 (0.00)	1.00–1.00	0.33	1.00 (0.00)	1.00–1.00	0.02	1.00 (0.00)	1.00–1.00	<0.001	1.00 (0.00)	1.00–1.00	<0.001
Male	[Reference]											
Female	1.03 (0.01)	1.01–1.05	0.00	1.05 (0.01)	1.03–1.07	<0.001	1.08 (0.02)	1.05–1.12	<0.001	1.02 (0.02)	0.97–1.07	0.44
Non-Hispanic White	[Reference]											
Non-Hispanic other	1.19 (0.02)	1.14–1.23	<0.001	1.48 (0.02)	1.42–1.54	<0.001	1.54 (0.03)	1.44–1.64	<0.001	1.37 (0.05)	1.23–1.51	<0.001
Non-Hispanic Black	1.22 (0.02)	1.18–1.26	<0.001	1.63 (0.02)	1.58–1.69	<0.001	1.83 (0.03)	1.74–1.92	<0.001	1.78 (0.04)	1.66–1.92	<0.001
Hispanic	1.37 (0.01)	1.33–1.40	<0.001	1.71 (0.02)	1.66–1.76	<0.001	1.58 (0.03)	1.50–1.66	<0.001	1.46 (0.04)	1.35–1.57	<0.001
No high school	[Reference]											
High school	1.13 (0.02)	1.09–1.16	<0.001	0.96 (0.02)	0.93–1.00	0.03	0.72 (0.02)	0.69–0.75	<0.001	0.61 (0.03)	0.57–0.65	<0.001
Some college	1.00 (0.02)	0.96–1.03	0.93	0.73 (0.02)	0.70–0.76	<0.001	0.51 (0.03)	0.48–0.54	<0.001	0.42 (0.04)	0.39–0.46	<0.001
College	0.89 (0.02)	0.86–0.92	<0.001	0.55 (0.02)	0.53–0.57	<0.001	0.35 (0.03)	0.33–0.37	<0.001	0.27 (0.04)	0.25–0.29	<0.001
Graduate school	0.78 (0.02)	0.74–0.81	<0.001	0.43 (0.03)	0.41–0.46	<0.001	0.22 (0.04)	0.20–0.24	<0.001	0.20 (0.06)	0.17–0.22	<0.001
Migration status	1.03 (0.02)	0.99–1.07	0.10	1.05 (0.02)	1.00–1.10	0.05	0.96 (0.04)	0.89–1.04	0.37	0.94 (0.06)	0.83–1.07	0.36
Family income (log)	1.18 (0.01)	1.16–1.20	<0.001	1.36 (0.01)	1.34–1.39	<0.001	1.67 (0.01)	1.63–1.72	<0.001	2.13 (0.02)	2.05–2.23	<0.001
Family income (log)^2^	0.98 (0.00)	0.98–0.99	<0.001	0.97 (0.00)	0.97–0.97	<0.001	0.95 (0.00)	0.94–0.95	<0.001	0.92 (0.00)	0.92–0.93	<0.001
County level characteristics												
Median household income (log)	0.86 (0.04)	0.79–0.93	<0.001	0.76(0.05)	0.69–0.84	<0.001	0.79 (0.07)	0.68–0.91	0.00	0.61(0.11)	0.49–0.75	0.97
Median age	1.01 (0.00)	1.10–1.02	<0.001	1.01 (0.00)	1.00–1.01	0.03	1.00 (0.00)	1.00–1.01	0.40	1.00 (0.01)	0.99–1.01	<0.001
Bachelor's degree or higher (%)	0.99 (0.00)	0.99–0.99	<0.001	0.97 (0.00)	0.98–0.98	<0.001	0.98 (0.00)	0.97–0.98	<0.001	0.98 (0.00)	0.97–0.98	<0.001
Population (10,000)	1.00 (0.00)	1.00–1.00	0.35	1.00 (0.00)	1.00–1.00	<0.001	1.00 (0.00)	1.00–1.00	0.16	1.00 (0.00)	1.00–1.00	0.72
Hispanic (%)	0.99 (0.00)	0.98–0.99	<0.001	0.97 (0.00)	0.97–0.98	<0.001	0.98 (0.00)	0.97–0.98	<0.001	0.97 (0.00)	0.96–0.98	<0.001
Non-Hispanic Black (%)	0.99 (0.00)	0.98–0.99	<0.001	0.97 (0.00)	0.97–0.97	<0.001	0.98 (0.00)	0.97–0.99	<0.001	0.97 (0.00)	0.96–0.98	<0.001
Non-Hispanic White (%)	0.99 (0.00)	0.99–0.99	<0.001	0.97 (0.00)	0.97–0.98	<0.001	0.98 (0.00)	0.97–0.98	<0.001	0.97 (0.00)	0.97–0.98	<0.001
State^a^												
Year^a^												
N (counties)						316						
N (individuals)						303,203						

a Included as dummy variables, not reported here.

**Table 5:** County level current operational spending on parks and recreations and binary self-rated health, by age and sex.

	Overall			Female adult			Male adult			Youth		
	OR (SE)	CI	*p*	OR (SE)	CI	*p*	OR (SE)	CI	*p*	OR (SE)	CI	*p*
Current operational spending (county-level, %)												
Parks and recreation	0.95 (0.01)	0.93–0.97	<0.001	0.95 (0.02)	0.91–0.98	0.00	0.95 (0.02)	0.92–0.99	0.02	0.96 (0.04)	0.88–1.05	0.42
Health	1.00 (0.00)	0.99–1.01	0.65	1.00 (0.01)	0.99–1.01	0.92	1.00 (0.01)	0.98–1.01	0.60	0.99 (0.02)	0.96–1.02	0.62
Welfare	1.01 (0.00)	1.00–1.01	0.11	1.01 (0.01)	1.00–1.02	0.08	1.00 (0.01)	0.99–1.01	0.81	1.01 (0.01)	0.98–1.03	0.50
Hospital	1.00 (0.00)	1.00–1.00	0.35	1.00 (0.00)	0.99–1.00	0.25	1.00 (0.00)	1.00–1.00	0.82	1.00 (0.00)	0.99–1.01	0.71
Demographics												
Age, years	1.09 (0.00)	1.08–1.09	<0.001	1.10 (0.00)	1.09–1.11	<0.001	1.11 (0.00)	1.10–1.12	<0.001	1.00 (0.02)	0.97–1.04	0.82
Age^2^	1.00 (0.00)	1.00–1.00	<0.001	1.00 (0.00)	1.00–1.00	<0.001	1.00 (0.00)	1.00–1.00	<0.001	1.00 (0.00)	1.00–1.00	0.12
Male	[Reference]											
Female	1.03 (0.01)	1.00–1.05	0.05							0.92 (0.04)	0.84–1.00	0.06
Non-Hispanic White	[Reference]											
Non-Hispanic other	1.20 (0.03)	1.14–1.27	<0.001	1.22 (0.04)	1.13–1.32	<0.001	1.14 (0.05)	1.04–1.24	<0.001	1.30 (0.09)	1.08–1.56	0.01
Non-Hispanic Black	1.39 (0.02)	1.34–1.45	<0.001	1.44 (0.03)	1.36–1.53	<0.001	1.32 (0.03)	1.23–1.41	<0.001	1.37 (0.07)	1.19–1.58	<0.001
Hispanic	1.13 (0.02)	1.08–1.17	<0.001	1.20 (0.03)	1.13–1.27	<0.001	0.99 (0.03)	0.93–1.06	0.85	1.34 (0.06)	1.18–1.52	<0.001
No high school	[Reference]											
High school	0.68 (0.02)	0.66–0.71	<0.001	0.64 (0.03)	0.61–0.68	<0.001	0.73 (0.03)	0.69–0.77	<0.001	0.87 (0.20)	0.60–1.28	0.49
Some college	0.56 (0.02)	0.54–0.59	<0.001	0.54 (0.03)	0.51–0.57	<0.001	0.60 (0.04)	0.56–0.64	<0.001			
College	0.44(0.02)	0.42–0.46	<0.001	0.42 (0.03)	0.40–0.45	<0.001	0.45 (0.04)	0.41–0.48	<0.001			
Graduate school	0.33 (0.03)	0.31–0.35	<0.001	0.31 (0.05)	0.29–0.35	<0.001	0.34 (0.05)	0.31–0.38	<0.001			
Migration status	0.94 (0.03)	0.87–1.00	0.06	0.97 (0.05)	0.88–1.08	0.62	0.97 (0.06)	0.87–1.09	0.61	0.95 (0.09)	0.80–1.13	0.57
Household income (log)	1.50 (0.01)	1.46–1.53	<0.001	1.51 (0.02)	1.47–1.56	<0.001	1.51 (0.02)	1.46–1.57	<0.001	1.42 (0.04)	1.32–1.52	<0.001
Household income (log)^2^	0.96 (0.00)	0.96–0.96	<0.001	0.96 (0.00)	0.96–0.96	<0.001	0.95 (0.00)	0.95–0.96	<0.001	0.96 (0.00)	0.96–0.97	<0.001
County-level characteristics												
Median household income (log)	0.87 (0.06)	0.78–0.99	0.03	0.94 (0.09)	0.79–1.11	0.44	0.90 (0.10)	0.75–1.09	0.88	0.54 (0.20)	0.36–0.80	0.46
Median age	1.00 (0.00)	0.99–1.00	0.40	0.99 (0.00)	0.99–1.00	0.29	1.00 (0.01)	0.99–1.01	0.02	0.99 (0.01)	0.97–1.01	<0.001
Bachelor's degree or higher (%)	0.99 (0.00)	0.99–0.99	<0.001	0.99 (0.00)	0.98–0.99	<0.001	0.99 (0.00)	0.98–1.00	0.28	1.00 (0.01)	0.98–1.01	0.74
Population (10,000)	1.00 (0.00)	1.00–1.00	0.43	1.00 (0.00)	1.00–1.00	0.66	1.00 (0.00)	1.00–1.00	0.02	1.00 (0.00)	1.00–1.00	0.38
Hispanic (%)	0.99 (0.00)	0.99–1.00	<0.001	0.99 (0.00)	0.99–1.00	0.05	0.99 (0.00)	0.99–1.00	0.02	0.98 (0.01)	0.97–0.99	<0.001
Non-Hispanic Black (%)	0.99 (0.00)	0.99–1.00	0.03	1.00 (0.00)	0.99–1.01	0.87	0.99 (0.00)	0.99–1.00	0.06	0.98 (0.01)	0.97–1.00	0.01
Non-Hispanic White (%)	0.99 (0.01)	0.99–1.00	0.00	1.00 (0.00)	0.99–1.00	0.22	0.99 (0.00)	0.99–1.00	0.02	0.98 (0.01)	0.97–1.00	0.00
State^a^												
Year^a^												
N (counties)	316			316			316			316		
N (individuals)	303,203			112,020			99,251			91,801		

a Included as dummy variables, not reported here.
